# A longitudinal high-risk study of adolescent anxiety, depression and parent-severity on the developmental course of risk-adjustment

**DOI:** 10.1111/jcpp.12279

**Published:** 2014-06-06

**Authors:** Adhip Rawal, Lucy Riglin, Terry Ng-Knight, Stephan Collishaw, Anita Thapar, Frances Rice

**Affiliations:** 1 Department of Clinical, Educational and Health Psychology, University College LondonLondon, UK; 2 Child and Adolescent Psychiatry Section, Institute of Psychological Medicine and Clinical Neurosciences, MRC Centre for Neuropsychiatric Genetics and Genomics, Cardiff University School of MedicineCardiff, UK

**Keywords:** Depression, anxiety, adolescence, reward, decision-making

## Abstract

**Background:**

Adolescence is associated with developments in the reward system and increased rates of emotional disorders. Familial risk for depression may be associated with disruptions in the reward system. However, it is unclear how symptoms of depression and anxiety influence the development of reward-processing over adolescence and whether variation in the severity of parental depression is associated with hyposensitivity to reward in a high-risk sample.

**Methods:**

We focused on risk-adjustment (adjusting decisions about reward according to the probability of obtaining reward) as this was hypothesized to improve over adolescence. In a one-year longitudinal sample (*N *=* *197) of adolescent offspring of depressed parents, we examined how symptoms of depression and anxiety (generalized anxiety and social anxiety) influenced the development of risk-adjustment. We also examined how parental depression severity influenced adolescent risk-adjustment.

**Results:**

Risk-adjustment improved over the course of the study indicating improved adjustment of reward-seeking to shifting contingencies. Depressive symptoms were associated with decreases in risk-adjustment over time while social anxiety symptoms were associated with increases in risk-adjustment over time. Specifically, depression was associated with reductions in reward-seeking at favourable reward probabilities only, whereas social anxiety (but not generalized anxiety) led to reductions in reward-seeking at low reward probabilities only. Parent depression severity was associated with lowered risk-adjustment in offspring and also influenced the longitudinal relationship between risk-adjustment and offspring depression.

**Conclusions:**

Anxiety and depression distinctly alter the pattern of longitudinal change in reward-processing. Severity of parent depression was associated with alterations in adolescent offspring reward-processing in a high-risk sample.

## Introduction

Adolescence is a period of biological, neurocognitive and social change (Patton & Viner, 2007) during which the prevalence of emotional problems, particularly depression, increases markedly (Thapar, Collishaw, Pine, & Thapar, 2012). The reward system which includes the striatum and its connections to subcortical dopamine pathways and prefrontal cortex develops substantially during adolescence (Ernst, Pine, & Hardin, 2006; Forbes & Dahl, 2005). There appear to be differences in the way that adolescents and adults process rewarding stimuli and their value. Specifically, adolescents show a heightened propensity to engage in potentially rewarding behaviours (i.e. are more reward-seeking) and this is likely to be related to a normative difference in the maturational timetable of limbic and prefrontal brain regions (Blakemore & Robbins, 2012; Casey, Jones, & Hare, 2008; Ernst et al., 2006; Richards, Plate, & Ernst, 2013). Key developments during adolescence may lie in the coordination of reward-seeking tendencies with regulatory processes (Steinberg, 2005) and in learning the reward and risk value of situations (Ernst & Paulus, 2005). Given the cooccurrence of changes to the reward system and increases in affective disturbance, adolescence is an important stage to study developments in reward-processing and relationships with depression and anxiety.

Results of behavioural studies, using a range of monetary reward-related paradigms suggest that adolescent depression is associated with a diminished sensitivity towards reward (Forbes, Shaw, & Dahl, 2007; Guyer et al., 2006; Jazbec, McClure, Hardin, Pine, & Ernst, 2005; Rawal, Collishaw, Thapar, & Rice, 2013). Specifically, that depressed adolescents are less reward-seeking compared to healthy adolescents particularly under conditions when the possibility of obtaining a reward is high. Neuroimaging studies also show diminished activity in reward-related brain regions in adolescents with depression during the processing of rewarding stimuli (Forbes et al., 2009). Moreover, results from two longitudinal studies suggest that as well as being present in currently depressed adolescents, this type of diminished reward-seeking exists prior to and increases risk for later depressive symptoms and disorder (Forbes et al., 2007; Rawal et al., 2013). However, a number of important questions remain.

First, on the basis that the integration of reward-seeking behaviour with regulatory processes is a key development during adolescence (Steinberg, 2005) it is unclear to what extent psychopathology influences the development of the ability to adjust decisions about reward according to the probability of obtaining a reward. Thus, adjusting reward-seeking behaviour in line with external contingencies (risk-adjustment) refers to the rational strategy of showing a greater propensity to seek reward when the probability of obtaining a reward is high compared to when it is low. Depressed adults show lower levels of risk-adjustment than healthy controls (Murphy et al., 2001). However, a study of adolescents that used the same reward decision-making task, failed to find alterations in risk-adjustment in currently depressed adolescents or in those that later developed depression (Rawal et al., 2013). Instead, that study reported reductions in risk-seeking behaviour in current and future adolescent depression only when the probability of reward was high (but not for overall risk-adjustment). One possible explanation for this discrepancy in results from adult and adolescent studies using the same task is that the alterations in risk-adjustment seen in adult depression may take some time to develop. Thus, it may be that depressive symptomatology is associated with aberrations in the developmental course of risk-adjustment. However, to our knowledge, there are no longitudinal studies addressing this question.

Second, it is well recognized that depression and anxiety show a high level of concurrent and sequential comorbidity and share risk factors in common (Rutter, Kim-Cohen, & Maughan, 2006). However, evidence suggests that the relationship between youth anxiety and reward-processing may be different from that in depression. For instance, two studies found no behavioural differences between adolescents with recent or current anxiety disorders and healthy controls in their reward sensitivity (Forbes et al., 2007; Rawal et al., 2013). Jazbec et al. (2005) showed that the performance of anxious youth was more adversely affected by the possibility of loss of reward than that of healthy controls. Forbes et al. (2006) also reported differing patterns of neural response during reward-processing in anxious compared to depressed adolescents. The aforementioned studies grouped a number of anxiety disorders together which is a common approach but could potentially obscure important differences. For instance, a comparison of neural response to reward anticipation in adolescents with social phobia, generalized anxiety and healthy individuals found a pattern of striatal hyperactivation that was specific to social phobia (Guyer et al., 2012). For anxiety, reward-processing may be altered in comparison to healthy control groups but that the processes involved may be different than those involved in depression, with enhanced sensitivity to the possibility of loss of reward as opposed to reduced motivation to obtain or work for rewards playing a role and results may vary for different types of anxiety.

Finally, there has been considerable interest in reward-processing as a behavioural intermediate phenotype for depression whereby familial/genetic risk influences this process prior to the manifestation of depression symptoms. Family studies have suggested that offspring of depressed parents show diminished activation in relevant brain regions when processing rewarding stimuli (Gotlib et al., 2010; McCabe, Woffindale, Harmer, & Cowen, 2012). Another way of further assessing this is to examine severity of parental depression among parents with a history of recurrent depression. This is known to contribute to variation in offspring clinical outcome (Hammen & Brennan, 2003; Mars et al., 2012) and may also be associated with alterations in reward-processing within a high-risk group. This requires investigation.

We examined longitudinal relationships between reward-processing, depression and anxiety and parent depression severity in a one-year repeated measures study of adolescents at familial risk of depression. We specifically focused on risk-adjustment as our measure of reward-processing given that this ability is hypothesized to develop during adolescence (Ernst & Paulus, 2005; Steinberg, 2005). This study therefore focuses on a different aspect of reward-processing from that examined in a previous study based on this cohort (Rawal et al., 2013). We differentiated between symptoms of generalized and social anxiety in our analysis to examine potentially heterogeneous effects of anxiety disorders (Guyer et al., 2012). We examined the following research questions:

1. Does risk-adjustment improve over time in this short-term longitudinal study?
2. How does adolescent anxiety and depression influence the development of adolescent risk-adjustment?
3. Does parent depression severity influence adolescent risk-adjustment and does it influence relationships between adolescent anxiety, depression and risk-adjustment?

## Methods

### Participants

Participants were part of a longitudinal study of parents with recurrent unipolar depression and their biological adolescent offspring (Early Prediction of Adolescent Depression; Mars et al., 2012). A history of recurrent depression in the parent was verified using the Schedules for Clinical Assessment in Neuropsychiatry (Wing et al., 1990). Exclusion criteria were diagnosis of bipolar disorder or a history of mania in the index parent, the child not living at home, or child IQ less than 50. One eligible child per household participated. If more than one child was present and willing to participate, the youngest eligible child was selected. Parents were recruited from primary care in South Wales, United Kingdom (78%); from a previous community study of recurrent unipolar depression (19%); and from advertisements in primary care (3%).

Reward task data were available at baseline and follow-up (average lag = 13 months, *SD *= 1.56). 197 adolescents completed the reward task at baseline. Two participants were excluded due to parental bipolar disorder. Of the remaining 195 participants, 175 (90%) completed the reward task at follow-up. Noncompletion at follow-up was due to a shortage of equipment (5), computer failure (1), time limitations (6), participant withdrawal (8).

### Psychopathology

#### Adolescent depression and anxiety

Adolescent psychiatric disorders (depressive disorders, anxiety disorders, eating disorders, conduct disorder, oppositional defiant disorder, ADHD, bipolar disorder and psychosis) were assessed at baseline and follow-up using the Child and Adolescent Psychiatric Assessment (CAPA; Angold et al., 1995). This is a semi-structured interview which provides an assessment of child psychopathology over the preceding 3 months. Interviews were conducted separately with the parent and child. Inter-rater reliability was excellent (average kappa child depressive symptoms = .93; generalized and social anxiety symptoms = .96). All cases meeting DSM-IV diagnostic criteria and subthreshold cases were reviewed by two child psychiatrists and diagnoses were agreed by clinical consensus. A disorder was considered to be present if a diagnosis was made based on either the parent or child interview (Angold & Costello, 1995). DSM-IV symptoms counts of major depressive disorder (maximum of 9), generalized anxiety (maximum of 14) and social anxiety (maximum of 4) were used in analyses.

#### Severity of parental depression

At each assessment phase, the Schedules for Clinical Assessment in Neuropsychiatry (Wing et al., 1990) were used to assess whether an episode of DSM-IV depression had occurred in the past month. A timeline of the parent's previous depressive episodes was compiled using a life history calendar approach where life events are used as markers to aid recall (Caspi et al., 1996). Parents were required to identify their worst two depressive episodes which were assessed in detail to ascertain severity and level of impairment. Impairment was assessed using the Global Assessment of Functioning (GAF) scale (American Psychiatric Association, [DSM-IV], 1994). Parents also reported on any periods of hospitalization. In accordance with previous criteria (Hammen & Brennan, 2003), a severe parental episode was defined by the presence of either severe impairment (GAF < 30) or hospitalization as part of the two worst episodes or at any of the study assessment phases.

### Reward-processing

The Cambridge Gambling Task (CGT) is a well-characterized reward task (Clark et al., 2008) consisting of eight blocks of nine trials. At the start of each block, participants receive 100 points and try to maximize points by betting on gambles involving two possible outcomes. On each trial, 10 coloured boxes (blue or red) of varying ratios (9:1, 8:2, 7:3, 6:4, 5:5) are presented on screen in pseudorandom order. In the first phase, the participant must decide under which colour the computer has hidden a token. In the second phase, the participant must bet a proportion of their points on the chosen colour. Possible bets of varying magnitude are offered in a sequence (5, 25, 50, 75, 95% of points), in 2.5 s increments. The hidden token's location is subsequently revealed. The amount of the bet is then added to (if correct) or subtracted from (if incorrect) the total score.

The second phase of the task yields two measures of reward-processing: 1) reward-seeking (the proportion of points gambled on trials where the more likely outcome is selected); and 2) risk-adjustment which assesses the linear effect of ratio on betting behaviour i.e. the extent to which participants adjust reward-seeking behaviour to changing context (Clark et al., 2008). Risk-adjustment is calculated using the equation (2a + b−c−2d)/average bet, where ‘a’ represents the mean bet in the 9:1 ratio, ‘b’ represents the mean bet at the 8:2 ratio etc. Analysis of betting behaviour was limited to trials where the participant selected the more likely outcome to maintain independence of reward-seeking and decision-making. Trials where the ratio of boxes was equal (5:5) were excluded from statistical analysis.

### Control variables

#### Puberty

Self-ratings of pubertal development were obtained at baseline (Petersen, Crockett, Richards, & Boxer, 1988). Adolescents rated the extent to which their bodies had changed on pubertal indicators (growth, body hair, skin, breast development (female only), voice and facial hair (male only)) on a 3-point-scale (‘not at all’ to ‘a lot’). Scores on the male version could range from 0–10, on the female version from 0–8. The raw score was divided by the total number of items before combining the male and female scores into one variable. We also classified individuals as prepubertal, early-pubertal, midpubertal, late-pubertal and postpubertal using a method similar to that described in Carskadon and Acebo (1993). The majority of the sample was classified as late-pubertal (31%) although scores ranged from prepubertal (9%) to postpubertal (18%).

#### Intelligence quotient

Full scale IQ was assessed using 10 subscales of the Wechsler Intelligence Scale for Children (WISC-IV; Wechsler, 2004).

### Procedure

Assessments were conducted in families’ homes. Parents and adolescents over 16 years provided written informed consent, younger children provided written assent. Ethical review and approval was provided by the Multi-Centre Research Ethics Committee for Wales.

### Statistical analysis

Repeated measures analysis of variance (ANOVA) was used to examine reward task performance at baseline and follow-up. Time (baseline, follow-up) and the ratio of coloured boxes (9:1, 8:2, 7:3 and 6:4) were within-subjects factors. Interactions were followed-up with paired *t*-tests at individual ratios. Pearson's *r* was used to examine associations between continuous variables and Spearman's rho between continuous and binary variables. To examine how adolescent anxiety and depression influenced risk-adjustment over time, cross-lagged models were fitted to the data using MPlus 7.11 (Muthen & Muthen, 1998). This approach tests for the direction of influence over time e.g. whether effects are unidirectional or bidirectional while controlling for the temporal stability of measures and the association between constructs at the initial assessment (Kenny, 2004). Models were estimated using manifest measures and all paths were tested simultaneously which produces fully saturated models with no degrees of freedom (goodness of fit indices therefore cannot be estimated). Models without adjusting for the control variables were initially fitted to the data. We then examined whether adjusting for control variables altered the pattern of longitudinal relationship between risk-adjustment and psychopathology by including IQ, puberty and gender in the model. The influence of parent depression severity was considered with comparisons of the magnitude of cross-lagged parameter estimates between risk-adjustment, depression and anxiety for offspring of parents with and without a severe episode. Models where the path was fixed to be equal across groups was compared to those where the path was allowed to vary between groups (severe vs. not severe) using a scaled chi-square difference test which provides an estimate of the statistical significance of parameter comparisons. The MLR estimator was used in MPlus (Muthen & Muthen, 1998) which provides maximum likelihood parameter estimates with standard errors and chi-square test statistics which are robust to nonnormality. Full information maximum likelihood estimation (FIML) estimation was used to treat missing data, which allows all sample participants to be retained in the path analyses. FIML uses all of the available information for each participant rather than deleting participants or imputing values (Schafer & Graham, 2002).

Table1 illustrates descriptive statistics (age, gender, IQ, pubertal status and psychopathology) and correlations with risk-adjustment for the 175 adolescents with longitudinal CGT data. There were no significant cross-sectional correlations between continuous measures of age or puberty and risk-adjustment. IQ was associated with better risk-adjustment at both baseline and follow-up, female gender with better risk-adjustment at follow-up. Younger age and female gender were associated with greater *improvement* in risk-adjustment (*r *=* *−.242, *p *<* *.001; rho = .150, *p *<* *.05).

**Table 1:** Descriptive characteristics of participants with longitudinal reward task data and correlations with risk-adjustment

	Baseline Mean (*SD*) or% (*N*) range	Follow-up Mean (*SD*) or% (*N*) range
Descriptive characteristics		
Age	13.63 (3.00) 10, 18	14.67 (2.01) 10, 19
% male:% female	44.6 (78): 55.4 (97)	**–**
IQ	97.67 (12.00) 69, 131	**–**
Pubertal status	.89 (.46) 0, 2	**–**
Depressive symptom count	1.80 (1.88) 0, 8	1.76 (1.82) 0, 9
Generalized anxiety symptom count	1.87 (2.55) 0, 14	1.80 (2.34) 0, 12
Social anxiety symptom count	0.23 (0.69) 0, 4	0.16 (0.62) 0, 4
Any DSM disorder	28.0 (49)	26.3 (46)
Any depressive disorder	7.4 (13)	5.1 (9)
Any anxiety disorder	12.6 (22)	10.9 (19)
Any disruptive disorder	9.1 (16)	8.6 (15)

Correlations	Risk-adjustment baseline	Risk-adjustment follow-up	
Control variables measured at baseline		
Gender	.022	.154*
Age	.09	−.136
Puberty	.047	−.008
IQ	.238**	.271***
Cross-sectional associations with psychopathology		
Depression	**−**.101	**−**.127^a^
Social anxiety	.092	.143^a^
Generalized anxiety	.109	.007
Longitudinal associations with psychopathology		
Depression	**−**.115	**−**.175*
Social anxiety	.163*	.231**
Generalized anxiety	**−**.128	**−**.085
Parent depression severity		
Severe episode (Y/N)	**−**.129^a^	**−**.158*

a *p *<.1

* *p *<* *.05

** *p *<* *.01

*** *p *<* *.001.

### Change in risk-adjustment

**Figure 1:**
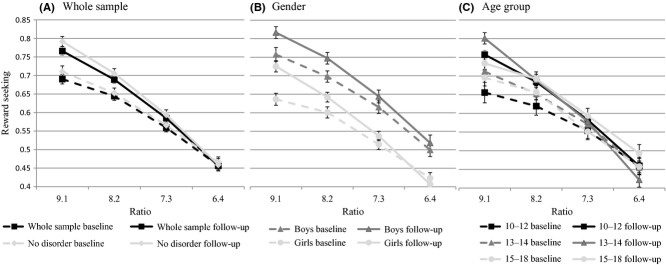
Descriptive statistics for reward-processing at baseline and follow-up. Dashed lines represent baseline levels of reward-seeking, solid lines represent follow-up levels. Reward-seeking = the proportion of existing points gambled when the more likely of the two possible outcomes (i.e. blue or red) is selected

Figure1 illustrates reward-seeking by each probability ratio at baseline and follow-up for the whole sample, those free from psychiatric disorder, males and females and by age-group tertiles (10–12; 13–14; 15–18 years) separately. Figure1 shows that at follow-up, reward-seeking increased at all probability ratios apart from the lowest probability ratio (i.e. when the probability of obtaining a reward (winning points) was lowest). Thus, the slopes of reward-seeking against ratio are steeper at follow-up than baseline (Figure1), which likely reflects increased risk-adjustment (i.e. adjusting reward-seeking behaviour in line with changing contingencies). This is borne out in results of repeated measures ANOVAs which showed an interaction between time and ratio (*F*(3,172)=9.29, *p *<* *.01, partial *η*^2 ^= .14). Follow-up analysis showed that participants were more reward-seeking at follow-up for ratios 9.1, 8.2 (*p *<* *.01) and 7.3 (*p *=* *.03) but not ratio 6.4 (*p *=* *.96), consistent with improved risk-adjustment. Indeed, there was a main effect of time on risk-adjustment (*F*(1,174)=17.37, *p *<* *.01, partial *η*^2 ^= .09). Excluding participants with DSM-IV disorders did not alter the pattern of results ((*F*(1,109)=14.91, *p *<* *.01, partial *η*^2 ^= .12). Figure1 shows that males were more reward-seeking than females and there was a trend for improvements in risk-adjustment to be greater for females as indicated by the interaction between risk-adjustment and gender (*F*(1,173)=3.11, *p *<* *.1; partial *η*^2 ^= .02). Figure1 shows that younger participants showed greater improvement in risk-adjustment and the interaction between age group and time was significant (*F*(2,172)=6.56, *p *<* *.01; partial *η*^2 ^= .01). We were able to evaluate the contribution of task familiarity to improved risk-adjustment by comparing 79 adolescents who completed the task for the first time at follow-up to 175 adolescents who completed the task for the second time. There were no differences between the groups on any key psychopathology or demographic variables with the exception of IQ which was lower in the ‘new’ group. The group completing the task for the second time at follow-up had higher levels of risk-adjustment when controlling for IQ (*F*(1,247) = 4.73, *p *=* *.03, partial *η*^2 ^= .02; *M *=* *1.19, *SE *= .06 vs. *M *=* *.96, *SE *= .09) although task familiarity did not entirely account for the improvement in risk-adjustment observed in the full sample as illustrated by the smaller effect size (partial *η*^2 ^= .02 vs. partial *η*^2 ^= .09 or partial *η*^2 ^= .14 in those free from disorder). Results therefore suggested that the pattern of change over this 1-year study was increased reward-seeking at high probability ratios at least partly reflected in greater risk-adjustment. We thus focused on examining how adolescent anxiety and depression influenced deviations from this pattern of development in reward-processing.

**Figure 2:**
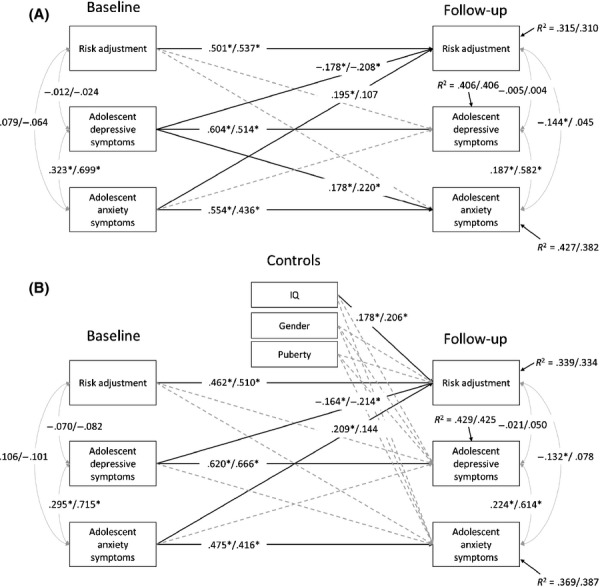
Cross-lagged models. Statistics separated by a slash refer to parameter estimates for social anxiety and generalized anxiety respectively

We next examined the longitudinal relationships between adolescent symptoms of depression, generalized anxiety and social anxiety and risk-adjustment using path analysis. All constructs showed moderate temporal stability and there was a significant initial correlation between both types of anxiety and depressive symptoms. In a cross-lagged model that included generalized anxiety, depression and risk-adjustment (Figure2, upper panel), there was a significant cross-lagged path between baseline depression and later risk-adjustment (*β *= −.208, *p *<* *.05) but generalized anxiety did not significantly influence later risk-adjustment (*β *= .107, *p *>* *.05). Thus, controlling for the stability of psychopathology over time, depression was associated with a reduction in later risk-adjustment although early risk-adjustment did not influence later psychopathology (depression or generalized anxiety). In a cross-lagged model that included social anxiety, depression and risk-adjustment, there were significant paths linking baseline depression and later risk-adjustment (*β *= −.178, *p *<* *.05) and baseline social anxiety and later risk-adjustment (*β *= .195, *p *<* *.05). Thus, both depression and social anxiety were associated with later risk-adjustment but in opposite directions. The lower panel of Figure2 illustrates that including IQ, puberty and gender in the model did not substantively alter the pattern of longitudinal relationships between adolescent depression, anxiety and risk-adjustment. To clarify the different associations of depression and social anxiety with reward-processing, we examined the effects of depression and social anxiety on betting behaviour at the highest (ratio 9:1) and lowest (6:4) probability ratios. Whereas depressive (*β *= −.159, *p *<* *.05), but not social anxiety symptoms (*β *= .050, *p > *.1) were associated with reductions in reward-seeking at ratio 9.1, the opposite pattern was evident at ratio 6:4 where social anxiety (*β *= −.124, *p < .1*), but not depressive symptoms (*β *= .060, *p *>* *.1) was negatively associated with the level of reward-seeking at follow-up. Thus, depressive symptoms selectively decreased reward-seeking at highly favourable reward contingencies (when the possibility for reward is greatest) and social anxiety symptoms selectively decreased reward-seeking at unfavourable reward contingencies (when the possibility for loss of reward is greatest). We examined whether the influence of adolescent depression on the developmental course of risk-adjustment was attributable to any specific symptoms. Appendix S1 shows that the symptoms of low mood, suicidality, psychomotor agitation/retardation and loss of energy were significantly associated with later reductions in risk-adjustment.

### How does parent depression severity influence the relationships between adolescent anxiety, depression and reward-processing?

Table1 shows that severe parental depression was correlated with adolescent risk-adjustment at follow-up. A repeated measure ANOVA revealed a significant interaction between ratio and severe parental depression (*F *=* *5.812, *p *<* *.001, partial *η*^2 ^= .093). Follow-up *t*-tests revealed that adolescents whose parents had severe depression were less reward-seeking at the high probability ratio 9:1 (*t *=* *2.454, *df *= 172, *p *=* *.015 baseline; *t *=* *3.275, *df *= 172, *p *<* *.001 follow-up) but not at the low probability ratio of 6:4 (*t *=* *.041, *df *= 172, *p *=* *.968 baseline; *t *=* *−.003, *df *= 172, *p *=* *.998 follow-up) suggesting reduced risk-adjustment. Indeed, there was a main effect of severe parental depression on adolescent risk-adjustment (*F *=* *4.654, *p *=* *.03, partial *η*^2 ^= .03). Including adolescent depression and social anxiety symptoms as covariates did not alter this result (*F *=* *4.154, *p *=* *.04, partial *η*^2 ^= .024).

When severe parental depression was included as a moderator of cross-lagged paths between adolescent depression, anxiety and reward-processing, it had no significant effect on the previously observed relationship between depression and later risk-adjustment (Δ*χ*^2^_(1) _= .355, ns) or social anxiety and later risk-adjustment (Δ*χ*^2^_(1) _= .037, ns). Neither did it influence the estimate between risk-adjustment and later social anxiety (Δ*χ*^2^_(1) _= 1.068, ns). However, there was a significant difference between the estimate for baseline risk-adjustment and later depression in the severe and nonsevere groups (Δ*χ*^2^_(1) _= 5.545, *p *<* *.05) where a significant inverse relationship was observed in the nonsevere group only (*β *= −.182, *p *<* *.05; *β = *.173, ns).

## Discussion

The reward system is thought to play an important role in affective and behavioural changes in adolescence (Ernst et al., 2006; Steinberg, 2005) and is involved in the development of adolescent depression. In this short-term longitudinal study, we observed improvements in risk-adjustment over time which indicates that participants improved in their ability to modify reward-seeking behaviour according to the probability of receiving a reward. Our first research question examined how depression and anxiety influence longitudinal development in risk-adjustment. Symptoms of social anxiety and depression had opposing effects on reward-processing while there was no significant influence of general anxiety. Specifically, depressive symptoms were associated with longitudinal reductions in risk-adjustment whereas social anxiety was associated with increases in risk-adjustment. Analysis of betting behaviour for the highest and lowest probability options showed that depressive symptoms predicted decreases in reward-seeking at high but not low probability ratios and that the opposite pattern was evident for social anxiety. Thus, depression symptoms were associated with little differentiation in betting behaviour across different ratios. These results support the growing literature on reward hyposensitivity in depression (e.g. Forbes et al., 2007, 2009; Rawal et al., 2013) and show that for adolescents who experience depression rewards appear to be a less potent motivator of approach behaviour. More specifically, they show that depression is associated with the development of a reward-seeking strategy where betting behaviour is not sufficiently guided by the probability of receiving a reward. We examined whether particular symptoms of depression were associated with this trajectory of lowered risk-adjustment. Symptoms of low mood, suicidality, psychomotor agitation/retardation and loss of energy showed longitudinal associations with lower risk-adjustment. Thus, these symptoms may be particularly important in accounting for alterations in reward-processing over time.

In contrast to the pattern of results seen for depression, social anxiety led to specific reductions in reward-seeking at less probable reward conditions. Reduced tolerance of uncertainty may produce low reward-seeking with unpredictable outcomes. For instance, one study reported enhanced striatal activation to reward anticipation in behaviourally inhibited adolescents only when they believed they were responsible for the outcome and presumably experienced greater uncertainty (Bar-Haim et al., 2009). We found evidence to suggest heterogeneous effects of different types of anxiety on reward-processing which is consistent with previous work on neural responses to reward anticipation (Guyer et al., 2012). Nonetheless, caution is warranted in drawing similarities between the present results and those of neuroimaging studies where group differences in task performance were not observed (Bar-Haim et al., 2009; Guyer et al., 2012). These results will require replication but are supported by evidence indicating that different processes are likely involved in altered reward-processing in depression and anxiety.

We next examined the role of parent depression severity on adolescent offspring reward-processing and in the longitudinal relationships between adolescent anxiety, depression and risk-adjustment. Severe parental depression was associated with lower adolescent offspring risk-adjustment. Parent depression severity also moderated the longitudinal relationship between offspring risk-adjustment and later depression with a significant inverse association observed only in the group of adolescents whose parents had not had a severe episode of depression. These findings are preliminary but highlight the complexity that is likely involved in the intergenerational transmission of depression and related disorders.

The associations between depression and anxiety with later risk-adjustment were independent of the effects of gender, puberty and IQ. Less able individuals may have struggled with the cognitive demands of the task. However, the pattern of results did not change when controlling for IQ. We treated puberty as a control variable in analyses as opposed to examining whether puberty might be a modifier of pathways linking adolescent depression, anxiety and risk-adjustment. This study was not designed to detect puberty-related changes in reward-processing as illustrated by the wide age range and short time span between assessments. The focus on a sample at high-risk for emotional problems also means it is not clear to what extent the findings will generalize to other samples and what the pattern of findings would be.

## Conclusion

Over a 1-year period, we observed improvements in adolescent risk-adjustment. Symptoms of depression and social anxiety (but not generalized anxiety) predicted distinct deviations from this trajectory suggesting that emotional problems have heterogeneous effects on reward-processing. Severity of parental depression influenced adolescent risk-adjustment highlighting that complex processes are likely involved in the inter-generational transmission of emotional disorders and is in keeping with the possibility that reward-processing may be an intermediate phenotype that confers familial vulnerability to depression.

## Supporting Information

Appendix S1 Associations between different symptoms of depression and risk-adjustment.
