# Supplementary material for: A longitudinal high-risk study of adolescent anxiety, depression and parent-severity on the developmental course of risk-adjustment
Source: J Child Psychol Psychiatry. 2014 Jun 6;55(11):1270–8. doi: 10.1111/jcpp.12279 (PMC4282404; doi:10.1111/jcpp.12279)
Supplement: Appendix S1 — Associations between different symptoms of depression and risk-adjustment. [file jcpp0055-1270-SD1.docx]

**Appendix S1: Associations between different symptoms of depression and risk-adjustment**

| **Baseline depressive symptoms** | **Risk-adjustment at follow-up** |
| --- | --- |
| Low mood | **-.146** |
| Irritability | -.100 |
| Loss of interest | -.092 |
| Change in appetite | -.074 |
| Sleep disturbance | -.064 |
| Agitation or retardation | **-.170** |
| Loss of energy | **-.143** |
| Worthlessness or guilt | -.079 |
| Inefficient thinking/indecisiveness | .024 |
| Suicidal thoughts/plans/behaviour | **-.150** |

Footnote: Associations significant at *p*<.05 are indicated in bold.
